# Correlation between microbial host factors and caries among older adults

**DOI:** 10.1186/s12903-021-01408-3

**Published:** 2021-02-04

**Authors:** Rakhi Mittal, Kai Soo Tan, Mun Loke Wong, Patrick Finbarr Allen

**Affiliations:** Faculty of Dentistry, National University of Singapore, Singapore, Singapore

**Keywords:** Streptococcus, Lactobacilli, Saliva flow, Buffering capacity, Dental caries

## Abstract

**Background:**

There is little knowledge about factors which may affect oral health among older adults. The objective of this study was to determine the relationship between *Streptococcus mutans* (MS) and Lactobacilli (LB) counts and caries among older adults.

**Methods:**

In this community-based observation study, 141 participants aged 60 years and above were recruited from the west district of Singapore. Alongside the clinical examination, saliva samples were collected to determine *Streptococcus mutans* (MS) and Lactobacilli (LB) counts, as well as to record salivary flow rate and buffering capacity of saliva.

**Results:**

Of the 141 participants, 63.8% were female and 94.3% were of Chinese ethnicity. The mean DMFT was 11.08 (s.d. 8.27). 9.9% of participants had at least one decayed tooth, 52.5% had minimum one missing tooth and 86.5% had at least one filled tooth. 67.4% had MS counts of ≥ 10^5^ while LB counts were ≥ 10^5^ for 48.2%. 83.7% had normal salivary flow or hypersalivation (> = 1 mL/min), the buffering capacity of the saliva was alkaline in 61% of the participants. Multivariate analysis showed that participants who had high MS counts were less likely to have a DMFT < 12 [OR (95% CI), 0.29 (0.11–0.77)] whereas participants who had high LB counts were less likely to have a DMFT ≤ 14 [OR (95% CI), 0.45 (0.20–1.002)].

**Conclusion:**

Our study showed a positive correlation between MS and LB counts and caries experience in older adults. The mean DMFT was on the low side in our sample despite having a relatively high MS count. This suggests that there are many other factors which vary according to host environment, physiological and biological conditions that may affect MS and LB counts in the oral cavity.

**Clinical relevance:**

Our study supports the knowledge that the aetiology of dental caries among older adults is a complex process and it would be wrong to consider caries as a same problem with the same solution for all age groups.

## Background

Population growth and aging has led to a dramatic increase in the burden of untreated oral conditions throughout the world in older adults [1]. Untreated cavitated dentine carious lesions in permanent teeth remained the most prevalent health condition across the globe in 2010, affecting 2.4 billion people [2]. The direct and indirect global economic impact of oral conditions may amount to more than US$442 billion [3]. Currently, older adults are found to have more of their natural teeth [4] which in turn puts them at greater risk of dental decay. Poor oral health condition of older adults can increase the risk to general health with compromised chewing and eating abilities [5]. Currently, ageing research primarily focuses on managing long-term conditions and improving disability and frailty in older age; there is little knowledge about factors which may prevent oral health problems in later life [6].

Dental caries is a multi-factorial, behaviourally mediated infectious disease that involves complex interactions between acid-producing bacteria, fermentable carbohydrates and many other host factors. The aetiology of dental caries involves a myriad of components which differs with age due to physiological changes in the human body [7]. *Streptococcus mutans* (MS) is one of the well-established etiologic agents for caries in both healthy and medically compromised individuals [8]. The predictive value of salivary levels of MS has been evaluated in many studies; however, the results are not consistent. Although some studies found a significant association between salivary levels of MS and subsequent caries onset [9] other studies revealed no clear-cut association between them [10, 11]. It has been shown that levels of salivary MS might not be the sole predictor for development of caries [12].

Some studies have indicated a relationship between Lactobacilli (LB) and dental caries [13, 14] whilst others found that caries lesions can develop in the absence of LB [15, 16]. The relationship between LB and dental caries, at least in humans, has not been proven to be cause-and-effect [16]. Various studies attempted to correlate changes in the composition of oral microflora with age; however there were certain limitations with the design of such studies including: the definition of older adults, their health status and, their level of dependency (housebound or institutionalised) [17].

Little is known about the microbial aetiology of dental caries in older adults (i.e., over 65 years of age) and there is no consensus as to which microbes cause oral diseases [18]. Thus, the predictive value of MS and LB counts in the saliva of older adults resulting in oral diseases remains unclear. Apart from increased colonisation with cariogenic bacterial species in the oral cavity, low salivary flow has also been linked to caries incidence [19]. Normal salivary flow protects the oral cavity, the upper airway and digestive tract and facilitates numerous sensorimotor phenomena [20]. With age, saliva flow rate reduces in older adults, and this may be influenced by medications and pathological changes in the salivary glands [21]. The main attributes of reduced salivary flow is self-reported mouth dryness (“xerostomia”) and increased incident dental caries.

Furthermore, few studies have demonstrated strong association between low caries levels and high salivary buffering capacity [22]. In the literature, larger quantities and faster rates of bacterial acid production have been consistently reported in caries active individuals than in caries-free individuals [7].

The influence of salivary flow and pH on dental caries were evaluated in children, adolescents and adults, whereas studies about the polymicrobial aetiology of dental caries in the older adults are incomplete, and the results remain controversial [23]. There is a little evidence available from epidemiological studies, and most studies lack statistical power [24]. Given the reducing prevalence of tooth loss with associated rise in carious teeth in an aging population, it is important to increase our understanding of the factors which moderate dental caries in older adults. The aim of our research is to assess the relationship between MS and LB levels, saliva flow rate, buffering capacity and coronal caries experience in older adults. The objective was to determine the association between microbial host factors and coronal and root caries in older adults.

## Methods

### Population and sample

A total of 141 community dwelling older adults were recruited from a senior activity centre located in the central west district of Singapore. The chosen sample for our study is adequate to investigate the objectives of the study. This study was part of Community Health Intergenerational (CHI) study [25]: an observational cohort study on ageing and mental health in community-dwelling older adults.

### Patient and public involvement

Participants in this study were not involved in the development of the study design or objectives. The research design and objectives were developed by the Principal Investigator (PI) and Co-Investigators (Co-Is) of this study and underwent review by a board of academic advisors affiliated to National University of Singapore Mind-Science Centre (NUS MSC). Presbyterian Community Services (PCS), a community partner, provided the study site (e.g., quiet rooms in Hannah Seniors’ Activity Centre (HSAC) to collect data). In addition, information about the study design and recruitment processes were shared with staff of PCS prior to data collection.

### Recruitment

Older adults 60 years and above were recruited from residences in the Toh Yi, Anak Bukit area in Singapore, and other areas within the district encompassed by a 10 km radius from the HSAC via door-to-door visits by research nurses and research assistants. Eligible individuals were also recruited onsite from HSAC, community centres, resident corners, senior activity centres and residences within the recruitment area—advertisement flyers were made available for visitors to the respective centres and by word of mouth. Interested individuals were invited to HSAC at their convenience. There were no specific exclusion criteria, except that participants should be between 60 and 99 years of age. Non-ambulant individuals who were keen to participate in the study gave their consent in their own homes. A member of the research team explained the study in detail and time was given to each individual to consider their participation in the study before signing the consent form.

### Dental clinical examination

The dental clinical examination was carried out by trained dentist in a quiet room located in HSAC. Clinical examination of all participants was carried out on a mobile dental chair. The dental examination included recording details of: (1) coronal status (decay, filled, missing due to decay or other reasons etc.) and root status (decay, filled, missing due to decay or other reasons etc.).

### Bacteriological examination

Alongside the clinical examination of the subject's dentition, saliva samples were also collected to determine MS and LB counts of the subjects, as well as to record salivary flow rate and buffering capacity of saliva. Generally, the determination of both MS and LB count increases accuracy of diagnosis and improves subsequent prognosis in the management of dental caries. Subjects were instructed to chew on paraffin wax for five minutes and spit out their stimulated residual saliva into a sterilised container. The pH of saliva was tested with a pH paper to determine its pH value against a calibrated chart. Secondly, the volume of stimulated saliva was recorded and the salivary flow per minute was subsequently calculated. After which, the saliva was applied onto Ivoclar Vivadent Inc® CRT kit (sneezing or coughing near the agar was avoided for the sake of test stability and mould prevention). Saliva was then allowed to flow on both surfaces of the CRT kit using a pipette without scratching the culture media. Thorough wetting was achieved by holding the carrier slightly oblique to prevent quick flow of saliva, before placing the agar immediately back into the test vial. A sodium bicarbonate tablet was added in the test vial, releasing carbon dioxide upon contact with moisture once the vial was tightly secured. The CRT kit was incubated at 37 °C for 48 h. The amounts of MS and LB were determined to be more than or less than 10^5^ colony forming units (CFU) using a chart provided by the manufacturer.

### Other data

Data on potential covariates were obtained from in-person interviews and included age, gender, ethnicity, education and monthly household income.

### Statistical analysis

The main outcome of this study was DMFT of older adults which was categorised as DMFT. Mean DMFT of coronal caries was 12 and mean DMFT for root caries was 14. To identify correlation between DMFT and other host factors including: age, gender, ethnicity, education, household income level, volume of saliva, buffering capacity of saliva, MS count and LB count, we first performed bivariate analysis. After bivariate analysis, we conducted multivariate analysis to predict potential factors associated with DMFT of study participants. *P*-value < 0.05 was considered significant. All statistical analyses were performed using SPSS version 24.

## Results

### Descriptive statistics

Table 1, shows that the majority of participants were between 60 and 75 years old, with a larger proportion of females (63.8%). The majority of the participants were of Chinese ethnicity (94.3%), 53.9% with monwthly household income of below S$5,000. The majority of the participants had at least a Primary education level (92.9%), with 31.9% having higher level or University level education.

**Table 1:** Shows socio-demographic characteristics of study population (N = 141)

Participants characteristics	N (%)
Age	
60–75 years old	112 (79.4)
More than 75 years	29 (20.6)
Gender	
Male	51 (36.2)
Female	90 (63.8)
Ethnicity	
Chinese	133 (94.3)
Non-Chinese	8 (5.7)
Monthly household income	
Below $5000	76 (53.9)
Above $5000	65 (46.1)
Education level	
No formal education	10 (7.1)
Primary passed and above	131 (92.9)

Table 2, shows data related to DMFT index of study population. The mean coronal DMFT was calculated to be 11.08 (Standard Deviation: 8.27). DMFT more than 12 was considered as high and DMFT less than 12 was considered low in this study. 9.9% of participants had at least one decayed tooth (DT), 52.5% had minimally one missing tooth (MT) and 86.5% had at least one filled tooth (FT). In total 34% of the study sample, had DMFT greater than 12 and the rest had DMFT less than 12. The mean root DMFT was calculated to be 13.8 (Standard Deviation: 0.48). 12.8% of participants had at least one decayed tooth (DT), 95.7% had minimally one missing tooth (MT) and 65.2% had at least one filled tooth (FT).

**Table 2:** Shows details about oral health characteristics of study population (N = 141)

Oral health characteristics	N (%)
Coronal DMFT	
DMFT < 12	93 (66.0)
DMFT ≥ 12	48 (34.0)
Decayed	14 (9.9)
Missing	74 (52.5)
Filled	122 (86.5)
Root DMFT	
DMFT ≤ 14	90 (63.8)
DMFT > 14	51 (36.2)
Decayed	18 (12.8)
Missing	135 (95.7)
Filled	92 (65.2)
MS count	
High (≥ 10^5^)	95 (67.4)
Low (< 10^5^)	46 (32.6)
LB count	
High (≥ 10^5^)	68 (48.2)
Low (< 10^5^)	73 (51.8)
Saliva volume	
Normal/hyper-saliva (> = 1 mL/min of saliva)	118 (83.7)
Hypo-saliva (< 1 mL/min of saliva)	23 (16.3)
Saliva buffering capacity	
Acidic	15 (10.6)
Neutral	40 (28.4)
Alkaline	86 (61.0)

*Mean DMFT of study population was 12

For bacterial count, 67.4% had high MS count of ≥ 10^5^ while 32.6% had < 10^5^ MS count. LB count was ≥ 10^5^ for 48.2% of the study sample, while 51.8% of the participants had < 10^5^ LB count.

Our results showed that 83.7% of participants had normal salivary flow or hypersalivation (> = 1 mL/min) while 16.3% had hypo salivation (< 1 mL/min of saliva). Buffering capacity of the saliva was mostly alkaline for the majority of the participants (61%), while 28.4% of them had saliva which was neutral and only 10.6% of them had acidic saliva.

### Correlates of coronal caries in the study population

#### Bivariate analysis

Table 3 shows bivariate correlation between DMFT and characteristics of study population. Significant correlation was found between coronal DMFT and MS count (*p* = 0.002) and LB count (*p* = 0.009). However, DMFT was not found significantly correlated with salivary volume (*p* = 0.15) and buffering capacity (*p* = 0.34). Root DMFT was found significantly correlated with LB count (p < 0.01).

**Table 3:** Shows bivariate correlation between DMFT and characteristics of study population (N = 141)

Study population characteristics	Coronal caries	Root caries
	*Bivariate analysis* (*p*-value)	Bivariate analysis (*p*-value)
Age	0.40	0.12
Gender	0.52	0.10
Ethnicity	0.17	-0.12
Education	0.08	-0.13
Household income	0.36	-0.16
MS count	0.002^a^	0.17
LB count	0.009^a^	< 0.01^a^
Saliva volume	0.15	0.96
Saliva buffering capacity	0.34	0.23

^a^Significant (*p* < 0.05)

### Multivariate analysis

Next, we performed multivariate analysis to predict potential host factors associated with coronal and root DMFT. After adjusting for covariates, we found that study participants who had high MS count were less likely to have a coronal DMFT < 12 [OR (95% CI), 0.29 (0.11–0.77)] whereas study participants who had high LB count were less likely to have root DMFT ≤ 14 (Table 4).

**Table 4:** Shows multivariate analysis between DMFT and host characteristics

Participants characteristics	Coronal caries		Root caries	
	Multivariate analysis	*p* value	Multivariate analysis	*p* value
	OR (95% CI)		OR (95% CI)	
Age				
60–75 years	1.15 (0.42–3.15)	0.77	1.22 (0.46–3.19)	0.68
More than 75 years	–		–	
Gender				
Male	1.11 (0.49–2.52)	0.78	1.55 (0.69–3.50)	0.28
Female	–		–	
Ethnicity				
Chinese	0.19 (0.02–1.83)	0.15	0.14 (0.01–1.44)	0.10
Non-Chinese	–		–	
Education				
No formal education	0.57 (0.11–2.80)	0.49	0.44 (0.08–2.26)	0.32
Primary and above	–		–	
Household income				
Below $5,000	0.69 (0.30–1.54)	0.36	0.54 (0.24–1.19)	0.12
> = $5,000	–		–	
Volume of saliva				
Normal/hyper (> = 5 ml)	1.51 (0.47–4.78)	0.48	0.45 (0.13–1.56)	0.21
Hypo (< 5 ml)	–		–	
Buffering capacity				
Alkaline (pH > 7)	1.13 (0.28–4.45)	0.85	1.70 (0.45–6.42)	0.42
Neutral (pH = 7)	1.11 (0.27–4.56)	0.88	1.42 (0.35–5.63)	0.61
Acidic (pH < 7)	–		–	
MS count				
High (> = 10^5^)	0.29 (0.11–0.77)	0.01*	0.67 (0.27–1.62)	0.37
Low (< 10^5^)	–		–	
LB count				
High (> = 10^5^)	0.58 (0.26–1.29)	0.18	0.45 (0.20–1.002)	0.05*
Low (< 10^5^)	–		–	

Outcome: DMFT, reference group: DMFT ≥ 12; *Significant (*p* < 0.05)

## Discussion

To our knowledge, this is the first study of dental caries conducted within a community-based research network in Singapore. The findings of our study emphasise that aetiology of dental caries is a complex process and it would be wrong to consider caries as a same problem with the same solution for all age groups [26]. Oral microbiome changes along with ageing [13] and many aspects of cultural diversity can influence oral hygiene routines, diet, health beliefs, and access to care, which may in turn affect oral health status [27].

Our study population had low DT scores and still MS count was found to be significantly associated with DMFT score. MS is considered to be a specific micro-organism that causes dental caries [7], there is evidence where MS count was low in presence of caries [28, 29]. In the present study, DT score is very low compared to MT and FT score, with majority of the study population having at least one filled tooth. For several years, amalgam filling has been considered as the main restorative material specifically for posterior teeth, however, at present all individuals prefer resin composite restorations because of their good aesthetics and adhesion properties [30]. It was been shown that MS colony forming unit (CFU) from resin composite group were significantly greater than amalgam restoration which means that physical and chemical characteristics of the restorative materials may affect MS count [31]. This validates the finding that MS count in oral cavity depends on various factors and not just on the presence of dental caries. Unfortunately, we didn’t record the type of restoration done in our study sample. It will be interesting to correlate the type of restorations with MS and LB counts in future studies.

Our study showed a significant association between root caries and LB count. This relationship should be interpreted with caution. The codes used for recording root status of study population did not include ‘missing due to caries’ which means that teeth which were missing were considered as missing whereas codes used for recording coronal status included ‘missing due to caries’ meaning teeth which were missing due to caries were considered as missing. Relationship between LB count and caries is still debatable, future studies should be done to explore and compare association between microbial host factors and coronal and root caries.

Bacteria in dental biofilms are regarded as important in initiation and progression of dental caries [32]. Previous studies showed association between dental caries and bacterial colonies in children, adolescents and adults, but studies about the polymicrobial aetiology of dental caries in older adults are incomplete or the results remain inconsistent [13]. In our study, LB count was found to be significantly associated with coronal caries experience in univariate testing, but it did not emerge as a significant variable in the multivariate testing or the final prediction model. At present, general opinion supports the concept that LB is not involved in the initiation of dental caries but more in the progression of deep enamel lesions [33]. Also, it has been reported that LB is not present in adults without active caries lesions [14]. In our study population, there were few active/untreated coronal lesions, and this highlights the need to further investigate the relationship between LB counts and carious activity in a population with higher likelihood of untreated caries, e.g., lower socio economic status (SES). This further emphasises the importance of a longitudinal study design to determine the prevalence of microorganisms in oral cavity of older adults.

It is interesting to note that the present study population showed a shift in DMFT scores from higher number of missing teeth to higher number of filled teeth in older adults. This could possibly be due to their relatively higher SES level, and thus more likely to have lower untreated decay and higher levels on restored teeth. These findings corroborates with previous longitudinal research in which there was a reduction in MT scores compared to FT scores in 65–74 year old senior citizens [34]. Furthermore, it has been proven that DT and MT components increased with decreasing income, while the FT component decreased [35]. The majority of our participants were educated, and significant proportion of participants had monthly household income above S$5,000. In Singapore, the median monthly household income from work grew by 3.0 per cent in nominal terms from $9,023 in 2017 to $9,293 in 2018, or 2.6 per cent in real terms. After accounting for household size, the median monthly household income from work per household member rose by 3.4 per cent in nominal terms from $2,699 in 2017 to $2,792 in 2018, or 3.0 per cent in real terms [36]. Hence, it can be inferred that our study population falls within the middle-higher income group.

This study characterised stimulated salivary flow and buffering capacity in a group of older Singaporeans. It has been documented that salivary flow varies according to the different parts of the mouth where it is measured and also between persons and across different biological situations [37]. Majority of our participants had greater than equal to 1 ml/min of saliva flow with an average of 1.87 ml/min. Our average value of saliva flow is higher than older adults in Japan 60 years old and above which has been reported to be 1.36 ml/min [38]. Interestingly, majority of our study participants had alkaline buffering capacity. This study used stimulated saliva to determine pH of saliva. Stimulation of saliva may affect salivary pH and the concentration of some constituents [39]. Chewing paraffin wax stimulate the parasympathetic response which increases saliva output from the parotid gland [40]. Therefore, stimulated saliva has a high buffering capacity [41], this theory may account for the high buffering capacity in the study population. Future studies may need to evaluate these findings in older adults.

Little is known about other factors apart from physiologic and biological changes responsible for saliva flow and buffering capacity. Salivary flow rate and buffering capacity are influenced by SES [37]. As previously stated, majority of our participants were educated and belonged to middle-higher income group, it has been consistently discussed in literature that individuals with better SES have better health conditions [42].

## Conclusion

This study found that MS and LB are an independent predictor of coronal and root caries respectively in older adults, but no significant association between caries and saliva flow and saliva buffering capacity was noted. Thus, one should interpret with caution an assessment of microbial and host factors to determine caries activity in older adults. Our study provides useful baseline data to inform future studies to look at potential factors which will yield more predictive value in determining caries risk in the older adults. Future longitudinal studies should be undertaken to explore this further, including saliva sample collection at different time points to explore determinants of caries experience in older adults.

## Data Availability

The data of this study may not be publicly available due to confidentiality agreements with the participants.

## Abbreviations

MS: Streptococcus Mutans
LB: Lactobacilli
CHI: Community Health Intergenerational
PI: Principal investigator
Co-Is: Co-investigators
NUS MSC: National University of Singapore Mind—Science Centre
PCS: Presbyterian Community Services
HSAC: Hannah Seniors Activity Centre
DMFT: Decayed Missing Filled Teeth
CRT: Caries risk test
CFU: Colony forming units
SES: Socio-economic status
